# Identification and characterization of multiple novel viruses in fecal samples of cormorants

**DOI:** 10.3389/fvets.2024.1528233

**Published:** 2025-01-09

**Authors:** Li Ji, Ran Zhao, Yifei Pei, Yijie Sun, Xiaoyi Sun, Likai Ji, Xiaochun Wang, Yuwei Liu, Quan Shen, Shixing Yang, Yan Wang, Wen Zhang

**Affiliations:** ^1^Department of Microbiology, School of Medicine, Jiangsu University, Zhenjiang, Jiangsu, China; ^2^Zhenjiang Mental Health Center, Zhenjiang, Jiangsu, China; ^3^Department of Prevention and Control, Xiamen Animal Disease Prevention and Control Center, Xiamen, China

**Keywords:** viral metagenomics, picornavirus, hepevirus, sequence alignment, phylogenetic analysis

## Abstract

**Introduction:**

Cormorants, as protected wild animals by the State Forestry Administration of China, have a broad distribution across China. Previous studies have shown that they can be infected with multiple viruses in the *Flaviviridae*, *Orthomyxoviridae*, *Paramyxoviridae*, and *Polyomaviridae* families. There is limited knowledge about the other viruses that cormorants may carry and infect.

**Methods:**

In this study, we employed viral metagenomics to identify novel viruses in the fecal samples collected from cormorants in Xiamen City, Fujian Province, China.

**Results:**

Two novel viruses were identified, including one novel picornavirus named Cormhepa01 and one novel avain hepevirus named CormhepaE. The genome of Cormhepa01 is 7,463 bp in length, which encodes a 2,260 aa polyprotien. Similar to other known picornaviruses, the conserved NTPase, proteinase, and polymerase motifs are presented in the 2C, 3C, and 3D proteins separately. Based on the phylogenetic analysis and amino acid sequence alignment, the CormhepaE may be assigned to a new picornavirus genus. The partial genome of CormhepaE is 6,546 bp in length. Compared with other avian hepatitis E virus strains, CormhepaE has multiple variable sites, which are distributed in motifs of the methyltransferase, helicase, and RdRp domains, separately. Based on the phylogenetic analysis, CormhepaE, together with another strain MG737712 isolated from sparrow, formed a new species of the *Avihepevirus* genus in the *Hepeviridae* family.

**Conclusion:**

We identified and characterized two novel cormorant viruses in this study. The findings of this study increase our understanding of the diversity of viruses in cormorants and provide practical viral genome information for the prevention and treatment of potential viral diseases affecting this species.

## Introduction

Viral emerging infectious diseases that threaten bird species are increasing at an unprecedented rate, exacerbating the decline in bird biodiversity and even causing species extinction (1). Besides, birds play a crucial role in the spread of infectious diseases, making them potential vectors for diseases that can affect livestock and human health. Birds are hosts of many zoonotic viral diseases, including influenza A virus in the family *Orthomyxoviridae*, Newcastle disease virus (*Paramyxoviridae*), West Nile virus (*Flaviviridae*), and Japanese encephalitis virus (*Flaviviridae*) in humans (2–4). Therefore, detecting potential new viruses in birds is the first step in maintaining bird health, protecting the stability of bird species, and maintaining the health of mammals and humans. Ordinary cormorants (*Phalacrocorax carbo*) are quite common and widespread in southern China and have long been domesticated by the people for fishing purposes. Due to close contact with humans, there is a risk of spreading the virus to infected individuals. To date, only viruses in the *Flaviviridae*, *Orthomyxoviridae*, *Paramyxoviridae*, and *Polyomaviridae* have been reported in cormorants. There is limited knowledge about the other viruses that cormorants may carry and infect.

Duck hepatitis A virus, a member of the *Avehepatovirus* genus within the *Picornaviridae* family, can cause highly pathogenic and contagious diseases that pose a significant threat to ducklings (5). *Picornaviridae* is a diverse family encompassing a wide variety of positive-sense single-stranded RNA (ssRNA) viruses, with a genome size of approximately 6.7–10.1 kb. Most picornaviruses contain a single open reading frame (ORF) flanked by 5′ and 3′ untranslated regions (UTRs). The *Picornaviridae* family currently contains 63 genera, among which picornavirus detected in birds and poultry not only comes from the *avehepatovirus* genus but also includes members of genera such as *aalivirus*, *avisivirus*, *grusopivirus*, *orivirus*, *crohivirus*, *sapelovirus*, etc. Picronaviruses are both heat and acid stable, which enable them to survive under extreme environmental conditions for easy transmission among birds. This resilience enhances their potential for transmission among animals through diverse pathways, including the fecal-oral route, direct contact with infected animal hosts, and potentially via invertebrate vectors.

Avian hepatitis E virus (HEV) is affiliated with the *Hepeviridae* family. Avian HEV is characterized by a small, non-enveloped virion and a 6.6-kb, single-stranded RNA genome. It has three ORFs. ORF1 is a polyprotein encoding putative functional domains of methyltransferase, papain-like cystein protease, helicase, and RNA polymerase. The capsid protein is encoded by ORF2, while ORF3 encodes a multifunctional phosphoprotein that is partly overlapped with ORF2 and is linked to viral morphogenesis and pathogenesis (6). Avian HEV has been identified as the causative agent of big liver and spleen disease (BLS), hepatitis splenomegaly syndrome (HSS), and hepatic rupture hemorrhage syndrome (HRHS) in chickens (7). Avian HEV is widespread in chicken flocks worldwide. The reported seropositive rates in chicken flocks are ranging from 27 to 95% in China, Korea, the USA, Spain, and Nigeria (8–12). The HEV-RNA prevalence in certain chicken farms is as high as 72–100% reported in China and the USA (13, 14). In recent years, various hepe-like viruses have been detected in birds from China, French Guiana, New Zealand, and Spain (15–18). However, due to limited data available, more species of HEV from different host ranges need further assignment.

Viral metagenomics has been proven to be a powerful method for exploring both novel and known viruses. The objective of this study is to identify potential infectious viruses in fecal samples collected from cormorants in Xiamen City, Fujian Province, China, using a viral metagenomic approach. Two novel viruses belonging, respectively, to the *Picornaviridae* and *Hepeviridae* families were identified in the present study. The findings of this study increase our understanding of the diversity of viruses in cormorants and provide practical viral genome information for the prevention and treatment of potential viral diseases affecting this species.

## Materials and methods

### Sample collection and preparation

In 2021, a total of 46 fecal samples from cormorants were collected using cotton swabs at the Xiamen International Horticultural Expo Garden, with bird expert identification during the collection. The swabs were immersed in 0.5 mL of Dulbecco’s phosphate-buffered saline (DPBS), followed by a 10 min vigorous vortexing. Post-centrifugation, the supernatant was transferred into 1.5 mL centrifuge tubes and stored at −80°C for further analysis. Ethical approvals were given by the Ethics Committee of Jiangsu University with the reference number 2018ujs18023. Sample collection was performed in accordance with the Wildlife Protection Law of the People’s Republic of China.

### Viral nucleic acid extraction and library construction

Every 9 to 10 swab extracts were collected together for a library, a total of 5 libraries were constructed, namely swab39, swab40, swab41, swab42, and swab43. For each library, a composite of 500 μL swab suspensions (50 to 55.6 μL of supernatant from each sample) was filtered through a 0.45 μm filter (Merck Millipore, Billerica, MA, USA) to eliminate bacterial and eukaryotic cell-sized particles. The filtrate was then subjected to a mixture of nuclease enzymes at 37°C for 90 min to degrade unprotected nucleic acids (19). Viral RNA and DNA were extracted using the QIAamp MinElute Virus Spin Kit (Qiagen, Hilden, NRW, Germany), and their concentrations were measured with the Qubit 4 (Invitrogen, Carlsbad, CA, USA) nucleic acid concentration sequencer. The RNA and DNA were stored at −80°C for further use.

The viral nucleic acid pool containing DNA and RNA viral sequences was subjected to RT reactions with SuperScript III reverse transcriptase (Invitrogen, CA, USA) using 100 pmol of a random hexamer primer. The RT reaction conditions are 25°C for 10 min, 50°C for 60 min, 85°C for 5 min, and 95°C for 2 min. Then, the reaction products were quickly removed and placed on ice for >2 min. The klenow enzyme (New England Biolabs, MA, USA) was used to generate the complementary chain of cDNA. The Klenow reaction conditions are 37°C for 60 min, 75°C for 20 min. Libraries were prepared with the Nextera XT DNA Sample Preparation Kit (Illumina, San Diego, CA, USA) according to the manufacturer’s protocol. A brief summary of the process includes adding connector primers and conducting 15 cycles of limited amplification.The library sequencing was completed by the Personalbio company using the NovaSeq Illumina platform with 250 base-paired ends with dual barcoding for each pool.

### Bioinformatics pipeline

The paired-end reads of 250 bp generated by the NovaSeq platform were decoded using Illumina’s proprietary software and processed through an in-house bioinformatics pipeline on a 32-node Linux cluster. Low-quality sequence tails were trimmed based on a Phred quality score threshold of 10. Adapters were removed using VecScreen with default settings. Bacterial reads were subtracted by mapping against the BLAST NT database with Bowtie2 v2.2.4 (20). Cleaned reads were *de novo* assembled with SOAPdenovo2 using a 63-mer size and default parameters (21). Assembled contigs and singlets were aligned to an in-house viral proteome database using BLASTx with an E-value cutoff of less than 10^−5^, where the viral BLASTx database was compiled using the fasta file of NCBI virus reference proteome (https://ftp.ncbi.nih.gov/refseq/release/viral/) (based on the annotation classification in the virus kingdom). Candidate viral sequences were further compared against an in-house non-virus non-redundant (NVNR) protein database to eliminate false positives, which was compiled from the NCBI NR database excluding sequences annotated with the virus taxonomy.

### Sequence alignment and ORF prediction

The pairwise comparison of viral amino acid sequences was conducted using SDTv1.2 software using the MUSLE parameter. Putative open reading frames (ORFs) in the genome were predicted using Geneious 11.1.2 software and the NCBI ORF finder.

### Phylogenetic analysis

The analysis of evolutionary relationships was carried out using nucleotide sequences or amino acid sequences identified in this study, with reference to the closest viral relatives determined by the best BLASTx hit, and representative members of related viral species or genera. Sequence alignment was conducted with MUSCLE in MEGA version X using the default settings (22). Phylogenetic trees were constructed using MrBayes v3.2.7 with the parameters “lset nst = 6 rates = invgamma.” This setting applied the GTR substitution model with gamma-distributed rate variation across sites and used a proportion of invariable sites (“GTR + I + Г”). Additionally, the parameter “prset aamodelpr = mixed” was employed to enable the program to use the ten built-in amino acid models. The maximum number of generations was set to be ten million, and sampling occurred at every 50 generations, with the first 25% of Markov chain Monte Carlo (MCMC) samples being discarded during burn-in. Convergence was confirmed when the standard deviation of split frequencies was below 0.01. Bootstrap values were assigned to each node.

### Nucleotide sequence accession number

The complete viral genome sequences identified in this study were deposited in GenBank under the accession numbers PQ583806 and PQ583807. The raw sequence reads from the metagenomic library were deposited in the Genome Sequence Archive (Genomics, Proteomics, and Bioinformatics 2021) at the National Genomics Data Centre (Nucleic Acids Res 2021), China National Centre for Bioinformation, Beijing Institute of Genomics, and Chinese Academy of Sciences (GSA: CRA010897).

## Results

### Viral metagenomic overview

The library generated a total of 15,872,272 raw sequence reads on the Illumina NovaSeq platform. The average GC content (GC %) was 48.1%. Bioinformatics analysis revealed that 533,790 sequence reads had the best match with viral proteins. Among them, 4,215 sequence reads were matched to the *Picornaviridae* family, while 1,153 sequence reads were matched to the *Hepeviridae* family.

### One novel picornavirus belongs to a new genus of the *Picornaviridae* family

**Figure 1:**
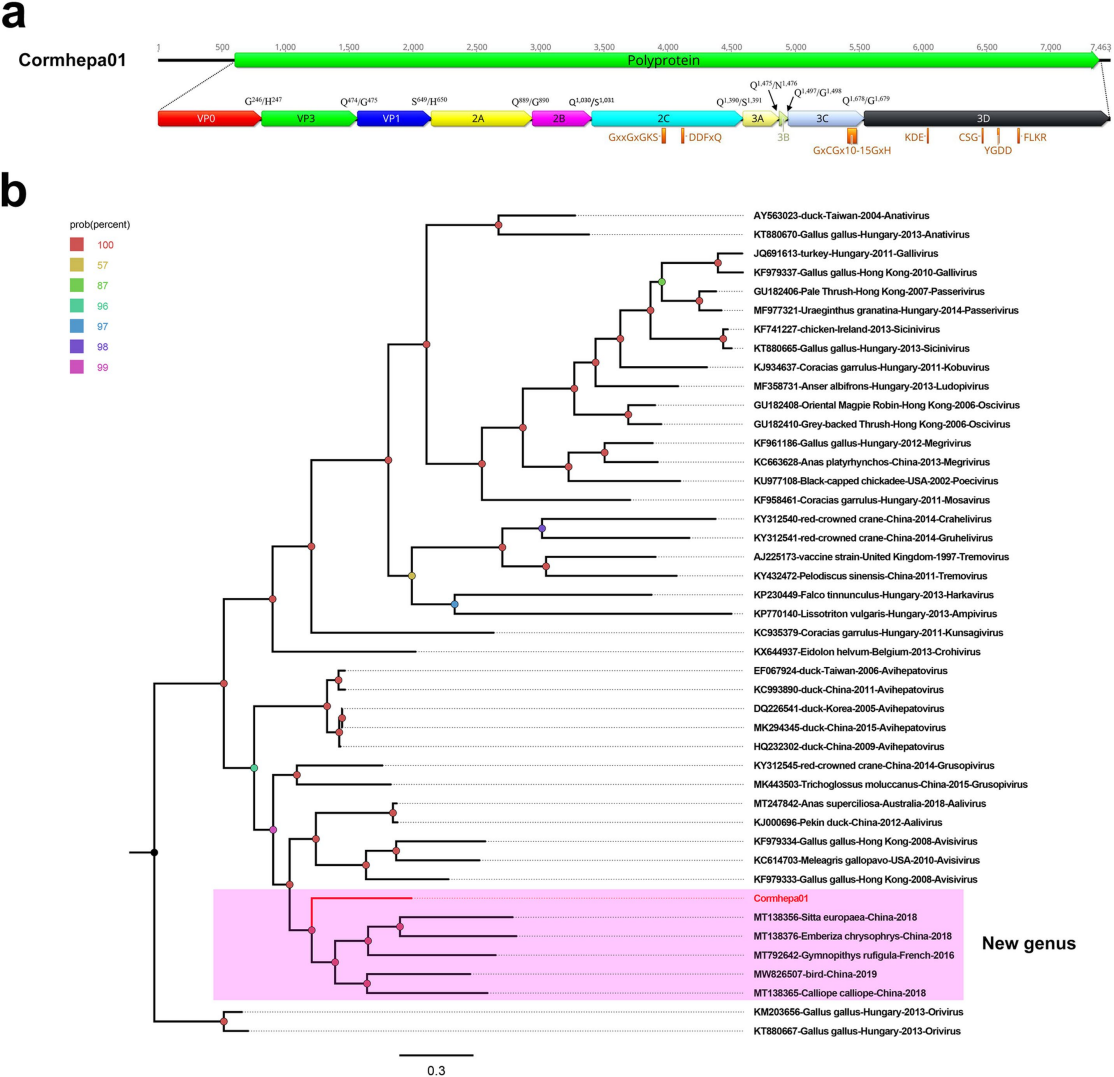
The genomic organization, conserved motifs, and phylogenetic analysis of the picornavirus identified in cormorants. **(A)** The genomic organization of Cormhepa01. The ORFs and viral encoding proteins of picornavirus were marked with different colors. The conserved motifs were also shown. **(B)**The phylogenetic analysis based on the 3D of picornavirus, which identified in this study, and other reference strains belonging to different genera of the *Picornaviridae* family. Cormhepa01 identified in this study were marked with red.

A total of 2,094,080 raw reads were generated from library swab41, among which, 5,368 reads matched to the *Avihepatovirus* genus with the mean coverage of 154.146. The complete genome of an avian hepatitis A-like virus was assembled and named Cormhepa01. The genome of Cormhepa01 is 7,463 in length, with the GC content of 42.2%. It contains a large ORF encoding a 2,260 aa polyprotien, flanked by a 602 bp of 5’ UTR and a 78 bp of 3’ UTR. Similar to other avian hepatoviruses, the polyprotein of Cormhepa01 can be cleaved into VP0, VP3, VP1, 2A-2C, and 3A-3D (Figure 1A). The P1 polypeptide of Cormhepa01 is composed of 649 aa and undergoes cleavage at the VP0/VP3 (G^246^/H^247^), VP3/VP1 (Q^474^/G^475^), and VP1/2A (S^649^/H^650^). The P2 polypeptide consists of 741 aa and contains three nonstructural proteins, including 2A (cleavage sites: Q^889^/G^890^), 2B (cleavage sites: Q^1,030^/S^1,031^), and 2C (cleavage sites: Q^1,390^/S^1,391^). As for other picornaviruses, the 2C protein of Cormcali01 displayed the NTPase motif ^1,198^GxxGxGKS^1,205^ and ^1,245^DDFxQ^1,249^, however, no NGPG motif was found in its 2A protein. The P3 polypeptide of Cormhepa01 is 870 aa in length and cleaved into four nonstructural proteins including 3A, 3B, 3C^pro^, and 3D^pol^ at cleavage sites 3A/3B (Q^1,475^/N^1,476^), 3B/3C (Q^1,497^/G^1,498^), and 3C/3D (Q^1,678^/G^1,679^), Conserved proteinase and polymerase motifs, including ^1,639^GxCGx_10-15_GxH^1,659^, ^1,828^KDE^1,830^, ^1,958^CSG^1,960^, ^1,995^YGDD^1,998^, and ^2,043^FLKR^2,046^ were presented in the 3C and 3D proteins (Figure 1A; Table 1).

**Table 1:** The detail information of cleavage sites, motifs, and protein functions of Cormhepa01 in this study.

Name of gene	Nucleotide sequence (bp)		Amino acid sequence (aa)		Predicted N-terminal cleavage site	Motifs and position	Predicted function of proteins
	position	length	position	length			
VP0	603–1,340	738	1–246	246	–		Capsid protein
VP3	1,341–2024	684	247–474	228	G/H (246/247)		Capsid protein
VP1	2025–2,549	525	475–649	175	Q/G (474/475)		Capsid protein
2A	2,550–3,269	720	650–889	240	S/H (649/650)		Primary polyprotein processing and Inhibition of cell growth
2B	3,270–3,692	423	890–1,030	141	Q/G (889/890)		Membrane associated
2C	3,693–4,772	1,080	1,031–1,390	360	Q/S (1,030/1031)	DDFxQ (1245–1,249) GxxGxGLS (1198–1,205)	ATPase
3A	4,773–4,895	255	1,391–1,475	85	Q/S (1,390/1391)		Membrane associated and Initiation of RNA synthesis
3B	4,896–5,093	66	1,476–149	22	Q/N (1,475/1476)		
3C	5,094–5,636	543	1,498–1,678	181	Q/G (1,497/1498)	GxCGxxxxxxxxxxxxxxGxH (1639–1,659)	Protease
3D	5,637–7,385	1749	1,679–2,260	582	Q/G (1,678/1679)	KDE (1829–1830) DxxxxD (1902–1907) CSG (1958–1960) YGDD (1995–1998) FLKR (2043–2046)	RNA-dependent RNA polymerase

**Figure 2:**
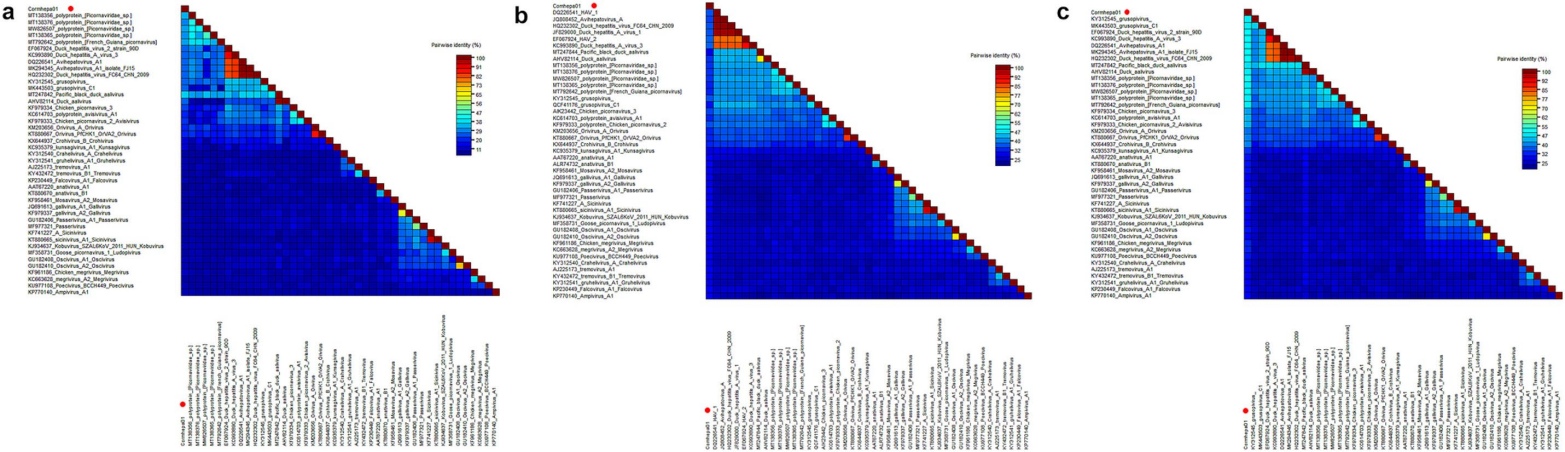
Pairwise comparison of amino acid sequences of one picornavirus identified in this study with the representative strains of different genera in the *Picornaviridae* family. **(A)** Pairwise comparison based on polyproteins. **(B)** Pairwise comparison based on P1. **(C)** Pairwise comparison based on the regions of 2C + 3CD. Cormhepa01 identified in this study was marked with a red solid circle.

Phylogenetic analysis was performed based on 3D of Cormhepa01 and viruses from other genus in the *Picornaviridae* family with avian origin, listed: aaliviruses, avisiviruses, grusopiviruses, oriviruses, crohiviruses, sapeloviruses, etc. The results showed that Cormhepa01 clustered with other unassigned picornaviruses (MT138356, MT138376, MT792642, MW826507, MT138365) detected from fecal samples of wild birds in China and France, forming a separate branch that was far away from other branches formed by known picornavirus genus (Figure 1B). To determine if they represented a new genus, amino acid sequence alignment was performed among polyprotein, P1, and “2C + 3CD” of Cormhepa01 with the same regions of other representative strains belonging to different genus of the *Picornaviridae* family (Figure 2). The polyprotein alignment result showed that Cormhepa01 shared the highest aa identities of 33.0% with the unassigned picornavirus MT138356 detected in wild birds in China, while having aa identities of <31.2% with other representative strains of picornaviruses. The P1 alignment result indicated that Cormhepa01 had the highest aa identities of 37.1% with a duck hepatitis A virus strain KC993890, while shared aa identities of 32.1 ~ 36.6% with other HAV strains. The alignment result based on the “2C + 3CD” region showed that Cormhepa01 shared the highest aa identity of 51.2% with the unassigned picornavirus MT138356 detected in wild birds in China and shared the aa identity of 49.3 ~ 49.5% with several duck hepatitis A virus strains. According to the International Committee on Taxonomy of Viruses (ICTV) classification for picornaviruses^1^ (accessed on October 28th, 2024), members of the genus of the *Picornaviridae* family should have significant divergence of the orthologous proteins exceeding 66% of P1^cap^ and 64% of 2C^hel^, 3C^pro^, and 3D^pol^. Combining with the phylogenetic analysis of Cormhepa01, it suggested that Cormhepa01, together with the other unassigned picornaviruses detected in birds or chickens may be assigned to a new picornavirus genus.

### One novel avain hepevirus belongs to a new species of the *Avihepevirus* genus

**Figure 3:**
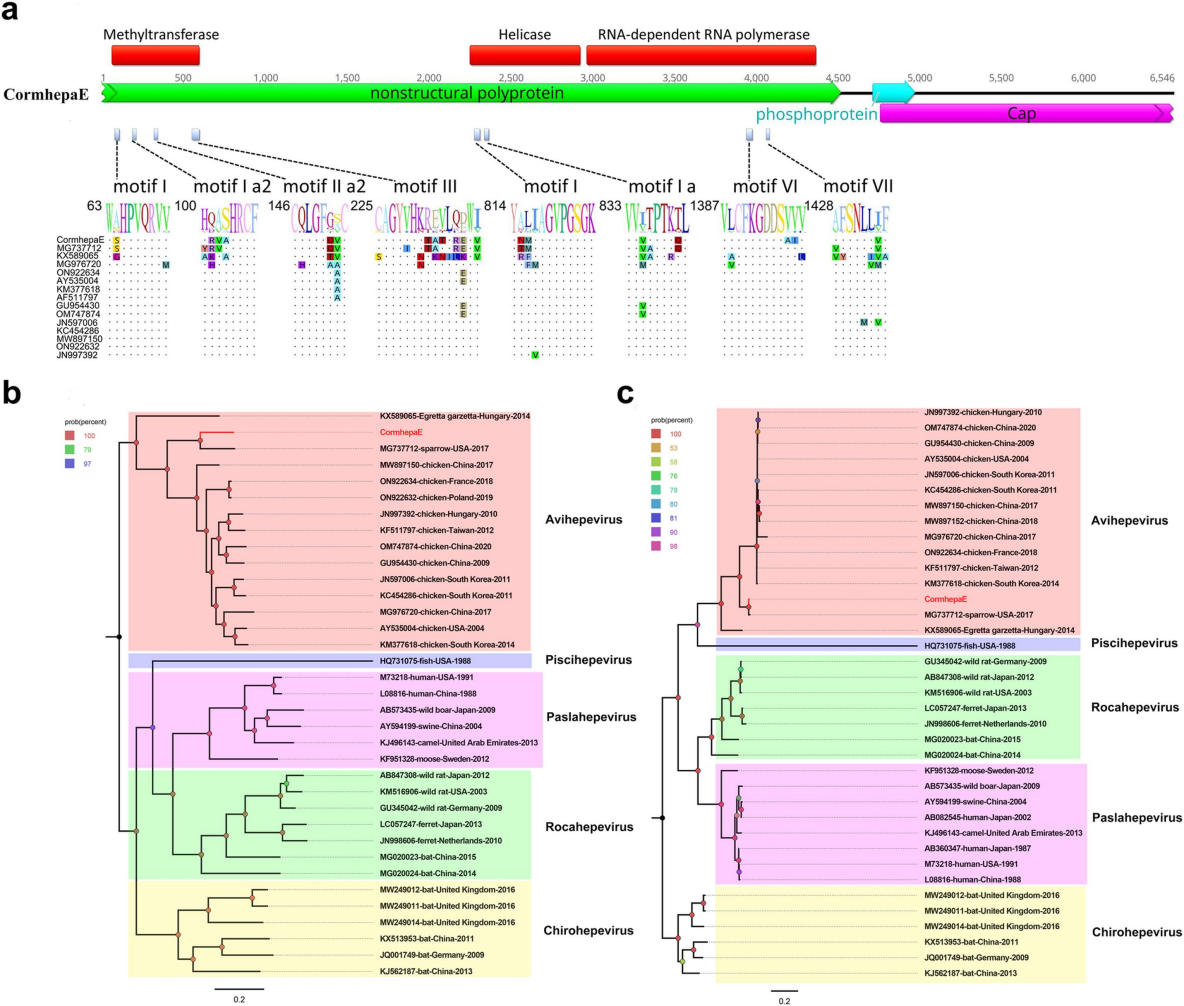
The genomic organization, mutant sites of methyltransferase, helicase, and RdRp domains, and phylogenetic analysis of hepevirus identified in cormorants. **(A)** The genomic organization and mutant sites in methyltransferase, helicase, and RdRp domains of one hepevirus identified in cormorants. Viral encoding proteins of CormhepaE were marked with different colors. **(B)** The phylogenetic analysis based on the complete genomic nucleotide of CormhepaE, which was identified in this study, and reference strains of different genera in the *Hepeviridae* family. The CormhepaE identified in this study was marked with red. **(C)** The phylogenetic analysis based on the capsid protein of CormhepaE and reference strains of different genera in the *Hepeviridae* family. The CormhepaE identified in this study was marked with red.

A total of 1,501,730 raw reads were generated from library swab43, of which, 1,023 reads matched to avain hepatitis E virus with the mean coverage of 34.611. The partial avian hepatitis E virus genome was assembled with a length of 6,546 bp and was named CormhepaE. CormhepaE contains three ORFs like other avian hepatitis E viruses, with partial nonstructural polyprotein and capsid protein and a full phosphoprotein. ORF2 encodes the partial nonstructural polyprotein of CormhepaE that consists of 1,504 aa, and it contains the methyltransferase, helicase, and RNA-dependent RNA polymerase domain with conserved motifs. Compared with other avian HEV strains, CormhepaE has multiple variable sites, which are distributed in motifs I, Ia2, IIa1, and III of the methyltransferase domain, motifs I and Ia of the helicase domain, and motifs VI and VII of the RdRp domain (Figure 3A). ORF2 encodes the partial capsid protein and consists of 596 aa, and it contains a glycosylation site (^256^NLS) that was reported in other HEVs. ORF3 encodes the phosphoprotein and consists of 86 aa like other HEVs.

**Figure 4:**
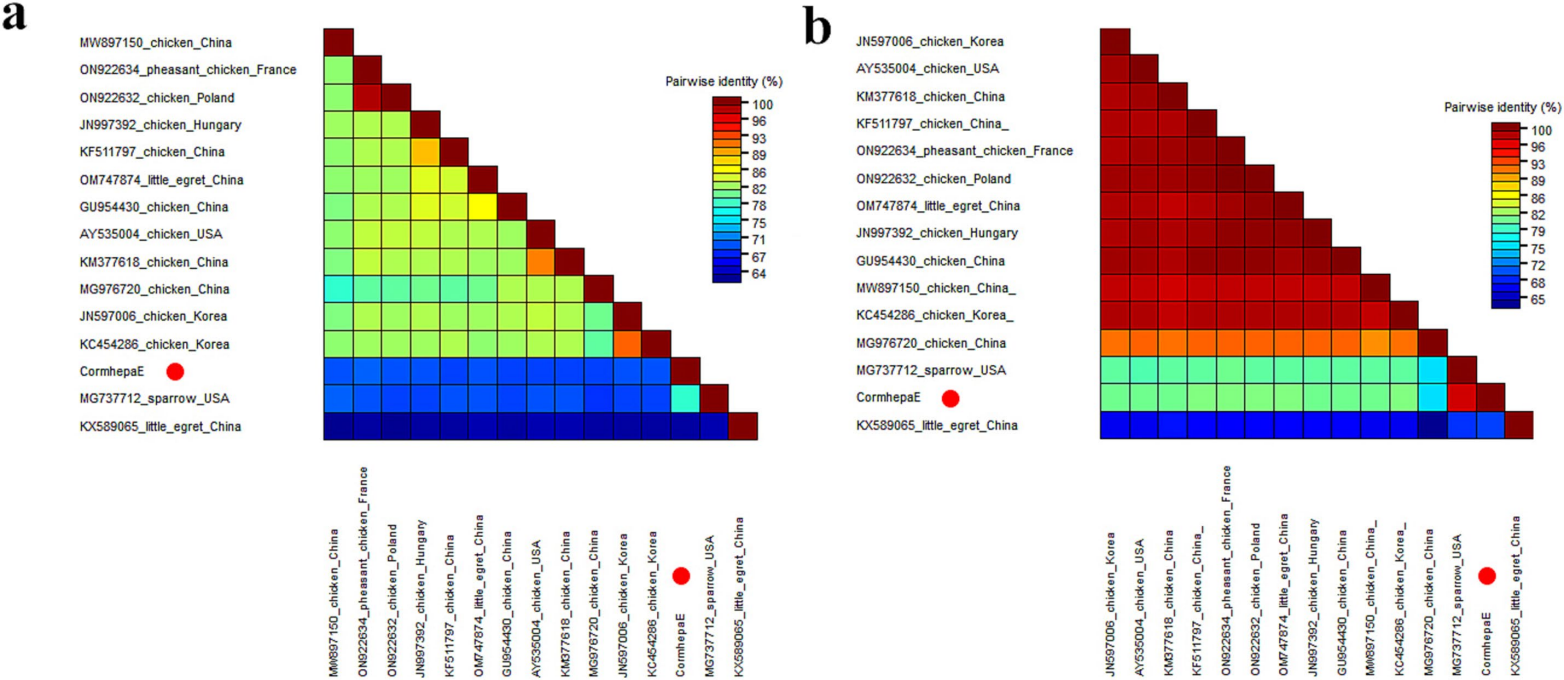
Pairwise comparison of nucleotide sequences and amino acid sequences of one hepevirus identified in this study with the representative strains of different genera in the *Hepeviridae* family. **(A)** Pairwise comparison based on complete genomic nucleotide sequences. **(B)** Pairwise comparison based on capsid proteins. CormhepaE identified in this study was marked with a red solid circle.

Phylogenetic analysis was performed based on the complete genomic nucleotide and capsid protein of CormhepaE and other HEVs from the *Paslahepevirus*, *Rocahepevirus*, *Chirohepevirus*, and *Avihepevirus* genere from the *Orthohepevirinae* subfamily and one HEV from fish origin as an outgroup. The results showed that CormhepaE clustered with one avian-like HEV detected in sparrows (MG737712) in the USA (Figures 3B,C). The partial nucleotides of CormhepaE were aligned with other AHEVs, results showed that it shared 68.8–77.7% nucleotide identity with other avian HEVs (Figure 4A). To determine if they represented a new species, the amino acid of capsid protein of CormhepaE was aligned against the same regions of other representative strains belonging to different species of the *Avihepevirius* genus. The result showed that CormhepaE shared the highest aa identity of 97.3% with the avian-like HEV detected in sparrows (MG737712) in the USA and less than 81.3% with other avihepeviruses that were detected in chicken and little egrets (Figure 4B). According to the International Committee on Taxonomy of Viruses (ICTV) classification for hepeviruses^2^ (accessed on October 28th, 2024), members of different species in the genus are phylogenetically distinct based upon analysis of ORF1 codon positions 1–450, ORF1 codon positions 971–1,692, and ORF2 codon positions 121–473, which suggested that CormhepaE and the strain MG737712 should be assigned to a new species of the *Avihepevirus* genus.

## Discussion

Bird populations carry a large number of different viruses. Healthy individuals possess significant viral diversity and may become natural hosts of infection. Cormorants are wild animals protected by the State Forestry Administration of China and are widely distributed in China. Previous studies have identified various viruses that can infect and cause diseases in this species. For instance, K. Nazerian and his coworkers first isolated a new herpesvirus from the blood of a nestling cormorant in 1973 (23). Krisztián Bányai and his coworkers identified a novel gamapolyomavirus in a died cormorant at the Zoo and Botanical Garden, Budapest (Hungary) in 2019 (24). Additionally, other viruses like cressdnavirus, microvirus, gyrovirus, and caudovirus were detected in the fecal samples of double-crested cormorant in the USA in 2021 by Arvind Varsani and his coworkers (25). In China, there are currently no reports of cormorant infection or virus carrying. Given this background, our study employed the viral metagenomic method to identify novel viruses in the feces of cormorants. Our findings identified two novel viruses in cormorants for the first time and classified them as new viral genus or species.

The *Picornaviridae* family currently consists of 158 species grouped into 68 genera. Picornavirus can infect various species including humans, mammals, and birds, causing diseases that affect various organs and systems, including the central nervous system, respiratory tract, gastrointestinal tract, heart, liver, pancreas, skin, and eyes (26–29). Duck hepatitis A virus (DHAV) can cause serious viral disease in young ducklings characterized primarily by hepatitis. DHAV is currently distributed globally and has the most important economic significance for all duck farms, as the potential mortality rate is high when the infection is not controlled (30). In addition to infecting ducks, DHAV can also host pigeons (31). In recent years, multiple avian hepatitis A-like viruses have been identified in birds from different countries and territories. In this study, we identified and characterized one novel avian hepatitis A-like virus from cormorant fecal samples. The finding of Cormhepa01 represents a significant contribution to our understanding of the genetic diversity and host spectrum within the *Picornaviridae* family. The near-complete genommic sequencing and subsequent phylogenetic analysis reveal that Cormhepa01 is closely related to those unclassified picornaviruses, formed independent branches and exhibited significant genetic divergence from other representative strains of different genera. The genomic structure and conserved motifs of Cormhepa01 are similar to those of the duck hepatitis A virus. It hinted that the novel hepatitis A-like virus was potentially able to cause disease in wild birds. Further epidemiological investigations are necessary to determine whether this virus can cause disease in cormorants and assess the potential for interspecies transmission.

*Avihepevirus* is a genus of the *Hepeviridae* family and is classified into two species, including Avihepevirus egretti and Avihepevirus magniiecur. Members of the Avihepevirus magniiecur species were mainly detected in chicken samples (14, 32, 33). As the pathogen of avain hepatitis E, the clinical disease in infected chickens has been referred to as big liver and spleen disease, and hepatitis-splenomegaly syndrome (34, 35). While the Avihepevirus egretti strain was first identified from cloacal specimens of healthy little egrets in Hungary (36). In the present study, one novel avain-like hepatitis E virus was first detected in fecal samples of cormorants and named CormhepaE. CormhepaE showed a closer relationship with the strain MG737712 isolated from sparrow fecal samples in the USA (37), while being relatively distant from other representative strains of the *Avihepevirus* genus based on the results of sequence alignment and phylogenetic analysis. According to the International Committee on Taxonomy of Viruses (ICTV) classification for hepeviruses (see text footnote 2), CormhepaE and the strain MG737712 can be classified into new species of the *Avihepevirus* genus. Currently, some novel avain-like hepatitis E viruses have been isolated from different birds (37–39). This prompted us to think that novel avain-like hepatitis E viruses can infect a broad range of hosts. Although we did not observe any significant clinical symptoms of bird infection with the virus during our sampling process, we cannot rule out the possibility of insufficient sampling leading to the undetected clinical diseases. Therefore, further experimental and epidemiological studies are needed to understand their pathogenicity and host species spectrum.

Although significant progress has been made in revealing novel viruses carried by wild cormorants in this study, there are still some limitations and shortcomings. Firstly, virus detection is based solely on fecal samples, which may not provide a comprehensive understanding of the distribution and infection status of the virus, as other tissues or body fluids may also carry these viruses. Secondly, despite the discovery of two new viruses (Cormhepa01 and CormhepaE), this study lacks further functional experiments to confirm their pathogenicity and transmission patterns under natural conditions. In addition, due to limitations in sampling location and time, the results may not fully represent the virus carrying situation of the entire cormorant population, especially considering that changes in geographic location and seasonal variations may affect the type and abundance of viruses. In summary, although this study provides crucial insights for further exploring the biological characteristics, ecological significance, and impact on host health of the novel virus, further follow-up work is needed to address existing shortcomings.

In conclusion, this study significantly advances our understanding of the viral diversity harbored by wild cormorants, identifying two novel viruses, Cormhepa01 and CormhepaE, for the first time. Cormhepa01, a member of the *Picornaviridae* family, exhibits significant genetic divergence and shares structural similarities with the duck hepatitis A virus, suggesting its potential to cause disease in wild birds. CormhepaE, a novel avian-like hepatitis E virus, belongs to the *Avihepevirus* genus of the *Hepeviridae* family and shows a close genetic relationship with a strain isolated from sparrows. These findings contribute to the knowledge of the genetic diversity and host range of these viruses. Future research should better understand the epidemiology, pathogenicity, and potential for interspecies transmission of these newly identified viruses.

## Data Availability

The original contributions presented in the study are publicly available. These data can be found in Genbank, accession numbers PQ583806-PQ583807.

## Footnotes

^1^ https://ictv.global/report/chapter/picornaviridae/hepatovirus

^2^ https://ictv.global/report/chapter/hepeviridae/hepeviridae/orthohepevirinae/avihepevirus
